# Comparing the Efficacy of Dynamic Neuromuscular Stabilization Exercises and Kegel Exercises on Stress Urinary Incontinence in Women: A Pilot Study

**DOI:** 10.7759/cureus.50551

**Published:** 2023-12-15

**Authors:** Kiran Sharma, Meena Gupta, Raju K Parasher, Jasmine Kaur Chawla

**Affiliations:** 1 Physiotherapy, Amity Institute of Health Allied Sciences, Noida, IND; 2 Physical Medicine and Rehabilitation, Venkateshwar Hospital, New Delhi, IND; 3 Physical Medicine and Rehabilitation, University of Delhi, New Delhi, IND; 4 Physiotherapy, School of Allied Health Sciences, Manav Rachna International Institute of Research and Studies, Faridabad, IND

**Keywords:** integrated spinal stabilization system, women, stress urinary incontinence, kegel exercise, dynamic neuromuscular stabilization

## Abstract

Background and objective

Stress urinary incontinence (SUI) is a prevalent condition affecting women of various age groups, significantly impacting their quality of life. To address this multifaceted issue, a comprehensive approach that goes beyond traditional pelvic floor exercises is needed. Dynamic neuromuscular stabilization (DNS) exercises, targeting the integrated spinal stabilization system, offer a promising alternative. Thus, this study aimed to compare the effectiveness of DNS exercises and Kegel exercises in managing SUI among women.

Methods

This single-blinded, pilot study involved 24 women aged 18-40 years with mild to moderate SUI. Participants were divided into DNS and Kegel exercise groups. Outcome measures included perineometer readings, electromyography (EMG) data, and the Urogenital Distress Inventory-6 (UDI-6). Statistical analysis compared baseline and 12-week data within and between groups, and rank-biserial correlation coefficient (r) as a measure of effect size in our study was calculated.

Results

At 12 weeks, the DNS group showed significant improvement in pelvic floor muscle strength compared to Kegel exercises (p = 0.005). Both groups had significantly enhanced pelvic floor muscle strength (p < 0.05). A significant change occurred for EMG average, EMG peak, and EMG maximum voluntary contraction (MVC) at 12 weeks (average p = 0.005; peak p = 0.001; MVC p = 0.009), with significant improvements in both groups (p < 0.05). For UDI-6, a significant difference emerged between the two groups at 12 weeks (p < 0.05), with significant improvements in both groups individually from baseline to 12 weeks (p < 0.05). The effect size "r" for all variables indicated a medium to large effect size, underscoring the substantial and significant impact of DNS exercises in managing SUI among women compared to Kegel exercises.

Conclusion

This study suggests that DNS exercises, emphasizing the coordinated activation of the diaphragm, abdominals, multifidus, and pelvic floor, may provide a more effective approach for managing SUI in women compared to traditional Kegel exercises.

## Introduction

Stress urinary incontinence (SUI) is a prevalent (4-35% prevalence rate) and often distressing condition that affects individuals, primarily women, across various age groups [1]. It is characterized by the involuntary leakage of urine during activities that increase intra-abdominal pressure, such as sneezing, coughing, running, or lifting heavy objects [2]. The impact of SUI extends beyond the physical symptoms, as it can significantly disrupt an individual's social life and personal well-being, ultimately compromising their overall quality of life [3].

Intriguingly, the prevalence of SUI appears to transcend age boundaries, affecting women not only as they age but also in their younger years [4]. This widespread occurrence underscores the importance of addressing this condition comprehensively. However, in many societies, particularly in countries like India, women often endure urinary incontinence silently, with reluctance to discuss their health issues openly [4]. Consequently, the reported prevalence rates might not accurately reflect the true scope of this problem as the reported statistics revealed that 20% of women consulted healthcare professionals for urinary incontinence, while 72% of those affected had been silently battling it for over a year, citing reasons such as unawareness and societal taboos as barriers to seeking help [4].

Understanding the causes of SUI reveals a complex interplay of factors. Vaginal deliveries, for instance, are associated with a heightened risk due to potential alterations in pelvic floor innervation, injury to the levator ani muscle, and damage to the endopelvic fascia from stretching or tearing. Moreover, vaginal births may lead to reduced mobility of the bladder neck, further exacerbating the risk of SUI [5].

The concept of the integrated continence system, encompasses deficits in the intrinsic urethral closure system, the urethral support system, and the lumbopelvic stability system [5]. These systems are interconnected through neural and endopelvic fascia connections and thus play a critical role in maintaining urinary continence [5]. The lumbopelvic region's control, in turn, relies on the coordination of various muscle groups, including the diaphragm, transversus abdominis, pelvic floor musculature, and lumbar multifidus [5]. These muscles regulate intra-abdominal pressure and the tension in the thoracolumbar fascia, influencing postural control [6].

However, addressing SUI is not as straightforward as concentrating solely on pelvic floor muscle strengthening. Emerging evidence suggests that the abdominal muscles also play a crucial role in achieving optimal results [7,8]. It has been asserted that pelvic floor muscle rehabilitation reaches its full potential when addressing the abdominal muscles in tandem [7]. It has been reported that pelvic floor muscle contraction led to increased activity in the abdominal muscles and vice versa, highlighting the potential benefits of abdominal muscle (transverse abdominis) training in SUI rehabilitation [7].

To tackle the multifaceted nature of SUI, a promising approach known as dynamic neuromuscular stabilization (DNS) may be introduced, which works on synergistic action of the entire core musculature [8-11]. DNS relies on developmental kinesiology, comparing the stabilizing patterns of individuals to those of healthy infants [12]. It targets the integrated spinal stabilization system, involving deep cervical flexors, diaphragm, transversus abdominis, multifidus, and the pelvic floor [8]. This system provides a stable foundation for purposeful activities by maintaining axial spine extension and centered extremity joint positions, thereby increasing intra-abdominal pressure sensed by the central nervous system [8]. DNS exercises aim to activate the spinal stabilizing system effectively through repetition, helping individuals regain control during various tasks [8].

Furthermore, dynamic core stability is crucial for optimal athletic performance and hinges on the precise coordination of the integrated spinal stabilization system and the regulation of intra-abdominal pressure [8-13]. This coordination is far more vital than simply building strength in isolated muscle groups as the muscles do not work in isolation [8-11,14].

The DNS method acknowledges that the core musculature functions as a cohesive unit in which the various components work together synergistically [15]. When one part of this system is weak or dysfunctional, it can have repercussions on the functioning of other core muscles [15,16]. DNS exercises are designed to address this interconnection by fostering comprehensive activation and strengthening of the entire core musculature, rather than targeting individual muscles in isolation [15,16].

By engaging in DNS exercises, individuals can train their core muscles to operate in harmony, providing stability and support to the spine, pelvis, and surrounding structures. As a result, this approach improves posture, enhances control over movements, and bolsters overall body stability [15]. DNS exercises contribute to the enhancement of core muscle strength by promoting synchronized activation, improving stability, and optimizing the performance of the entire core unit. By taking a holistic approach to core training, DNS exercises offer a comprehensive strategy for boosting core strength and stability, ultimately leading to improved overall physical function and performance. While DNS has been effectively utilized in various conditions, such as sports injuries, cerebral palsy, hemiplegia, and other diverse ailments [16-22], there is currently no research investigating the impact of DNS exercises on SUI.

Considering the intricate connections between the pelvic floor and core musculature, it becomes evident that a comprehensive approach to treating SUI is warranted. Thus, this study seeks to evaluate the efficacy of a DNS exercise program in comparison to a traditional pelvic floor exercise program for women dealing with SUI.

## Materials and methods

The study commenced after obtaining approval from the Institutional Review Board of Amity Institute of Health Allied Sciences (AUUP/IEC/2021-Jan/03) and was registered in the Clinical Trial Registry, India under the reference CTRI/2021/09/036247. It was designed as a single-blinded (participants were blinded), single-center randomized controlled trial. We recruited a convenient sample of 24 female participants (12 in each group) [23]. Participants were eligible if they were females aged 18-40 years, married, diagnosed with mild to moderate SUI, at least one-year post delivery, and medically and physically fit for assessment and physiotherapy. Exclusion criteria included continuous urinary leakage, current urinary incontinence drug therapy, pelvic prolapse > stage I, pregnancy, vaginal/urinary tract infections, menstruation during the examination, presence of tumors/fractures/acute inflammatory diseases, current estrogen treatment, and use of anticholinergics, antidepressants, or serotonin-affecting substances [24]. All the participants were recruited by convenience sampling after being referred and diagnosed by a gynecologist.

Randomization and allocation of participants

Participants who met the inclusion criteria and were screened for exclusion were assigned to one of the treatment groups in a 1:1 ratio. A random number table, generated by a statistician, was used to allocate participants to their respective groups. Subsequently, an allocation plan was meticulously documented in sequential order and securely sealed within opaque envelopes. During the allocation process, an independent individual, unrelated to the study, was responsible for opening the envelope to disclose the assigned group. This unbiased procedure determined whether participants were allotted to either the DNS or Kegel exercise group.

Procedure

**Figure 1:**
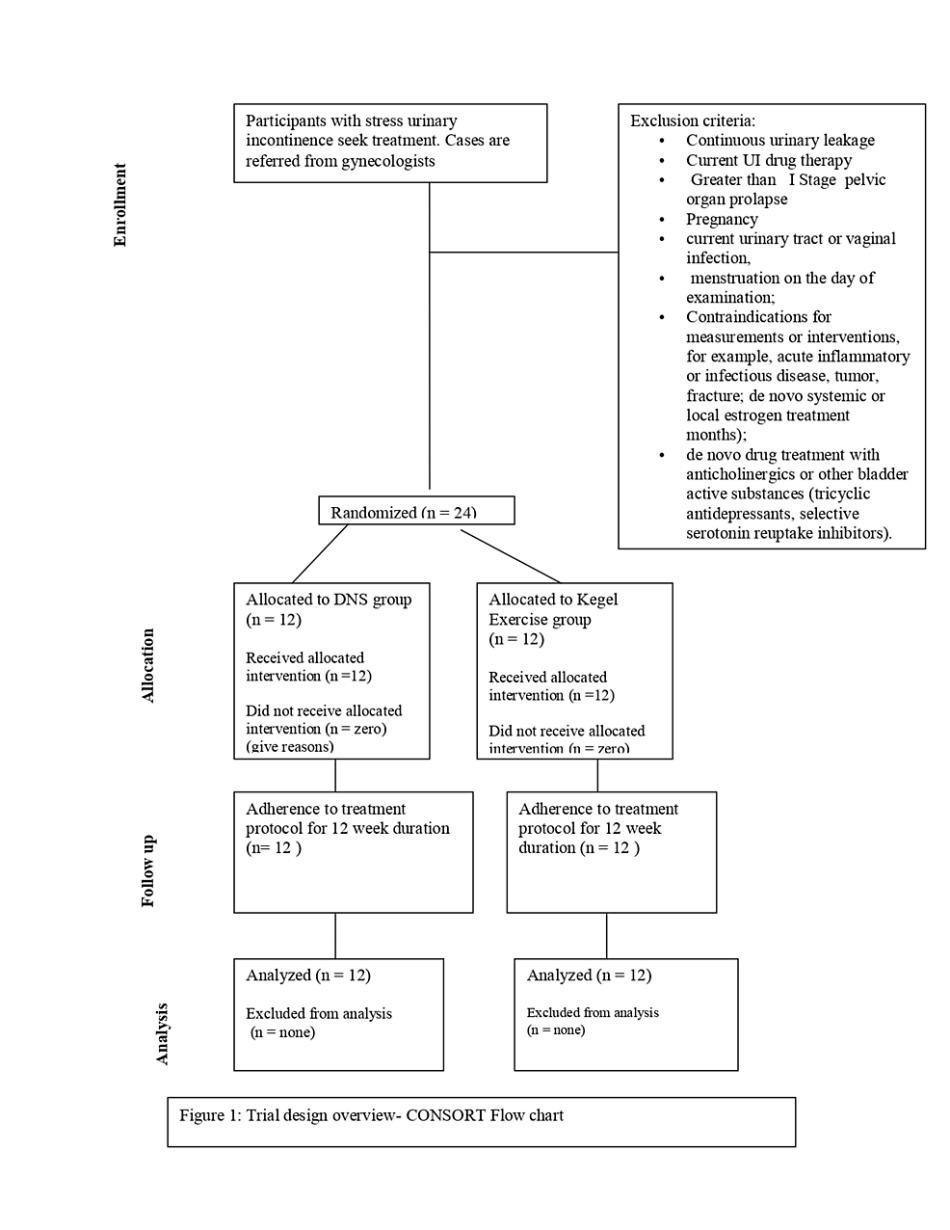
Trial design overview - CONSORT flow chart DNS: dynamic neuromuscular stabilization; CONSORT: Consolidated Standards of Reporting Trials.

Baseline measurements for outcome measures included pelvic floor muscle (PFM) strength (perineometer), electromyography (EMG) of PFM, and quality of life (Urogenital Distress Inventory-6 (UDI-6)). These measurements were repeated after a 12-week intervention period. The Consolidated Standards of Reporting Trials (CONSORT) flowchart, presented in Figure 1, provides a visual representation of the participant enrollment and progression throughout the study, illustrating their inclusion, allocation, follow-up, and analysis stages.

The experimental group followed a four-phase exercise protocol (detailed in Table 1), while the control group performed PFM contractions, holding each contraction for 10 seconds, with 10 repetitions per set and three sets in total, with one minute of rest between sets [24]. According to the DNS principles, for correct activation of the integrated spinal stabilization system, the abdominal muscles should not only expand in the caudal direction but should expand in all directions, i.e., the posterior and lateral as well. Thus, the investigator should palpate the medial to anterior superior iliac spine anteriorly and the posterolateral aspect of the abdominal wall below the lower ribs from behind to assess the correct expansion of the entire abdominal wall [16]. The exercises for both groups were carried out under the supervision of the investigator in the clinical setting. They were instructed not to perform the exercises at home.

**Table 1:** Dynamic neuromuscular stabilization exercise protocol for the rehabilitation of stress urinary incontinence

PHASE	DURATION (weeks)	PATIENT’S POSITION	INVESTIGATOR’S POSITION	INSTRUCTIONS	REPETITIONS	SETS
I	3	1. Crook lying	At the patient’s side, palpating the transverse abdominis muscle (medial to anterior superior iliac spine)	Contract the pelvic floor slightly like holding urine, and while maintaining this, inhale so that your abdominal wall expands against the therapist’s fingers, while maintaining this expansion, exhale out and then keep breathing normally	10	3
		2. Prone	The posterolateral aspect of the abdominal wall below the lower ribs from behind	Same as the previous exercise	10	3
IIa.	1.5	1. Supine with legs supported on a stool	Palpating the abdominal muscle medial to anterior superior iliac spine	Same as Phase I	10	3
		2. Quadruped	The posterolateral aspect of the abdominal wall below the lower ribs from behind	Same as Phase I	10	3
IIb	1.5	1. Supine with legs supported on gym ball	Palpating the abdominal muscle medial to anterior superior iliac spine	Same as Phase I	10	3
		2. Quadruped with knees supported on an unstable surface	The posterolateral aspect of the abdominal wall below the lower ribs from behind	Same as Phase I	10	3
III	3	Quadruped to heel sitting	Sitting behind the patient	Ask the patient to come in a heel sitting position and maintain the spine in neutral along with the abdominal and pelvic floor contraction while the therapist will resist the same in the first part, and in the second part, the patient will try to do heel sitting while the therapist’s force will overpower the patient’s force and movement will occur cranially in the direction of therapist’s force	10	3
IV	3	1. Sagittal stabilization with hip abduction	Palpating the abdominal muscle medial to anterior superior iliac spine	Contract the pelvic floor slightly like holding urine, and while maintaining this, inhale so that your abdominal wall expands against the therapist’s fingers, while maintaining this expansion, exhale out and then keep breathing normally, while maintaining this, ask the patient to take one hip in abduction while keeping the other hip in neutral (starting) position. Repeat the same on the other side	10	3
		2. Sagittal stabilization with hip flexion	Palpating the abdominal muscle medial to anterior superior iliac spine	Contract the pelvic floor slightly like holding urine and while maintaining this, inhale so that your abdominal wall expands against the therapist’s fingers, while maintaining this expansion, exhale out and then keep breathing normally, while maintaining this, ask the patient to take one hip in flexion while keeping the other hip in neutral (starting) position. Repeat the same on the other side	10	3

Outcome measures

In the completed study, the outcome measures encompassed changes in perineometer values, average, peak, and maximum voluntary contraction (MVC) of PFM as measured by EMG, and the impact of urinary incontinence on quality of life by using UDI-6 after the 12-week treatment period.

Perineometer

The device (Bionics Perineometer Analogue, Mumbai, India) consisted of a conical vaginal insert linked to a handheld microprocessor for measuring pressure in mmHg upon compression of the insert. Participants assumed a crook-lying position, and the perineometer was inserted into the vaginal canal until the compressible portion extended beyond the hymeneal ring. Baseline pressure readings were recorded, and participants were instructed to exert maximum effort to contract their PFM for two to three seconds, completing three consecutive squeezes with a one-minute rest interval. The peak of these three contractions represented their maximum perceived strength. Careful observations and a pressure biofeedback device were employed to ensure a neutral spine position and prevent elevated perineometer readings due to excessive intra-abdominal pressure. The vaginal insert was covered with a condom to ensure safe and hygienic use by multiple participants. The perineometer demonstrated high reliability, indicated by an intraclass correlation coefficient (ICC) of 0.95 [25].

EMG

EMG data were gathered using the NeuroTrac MyoPlus2A version 11.1 device (Hampshire, UK). A pear-shaped intravaginal sensor with stainless steel electrodes was gently inserted into the vagina while patients were in a supine lithotomy position. A reference electrode was placed on the right anterior superior iliac spine. Prior to examination, participants received training to ensure accurate contraction of their PFM. PFM strength was evaluated by recording the average scores from three maximum contractions. The EMG values were documented in terms of average (EMG average), peak (EMG peak), and maximum voluntary contractions (EMG MVC), with measurements in microvolts for EMG average and EMG peak (µV) and in percentage (%) for EMG MVC. An automated protocol software offered on-screen instructions and voice guidance, indicating when to contract and relax the PFM, following a pattern of five-second work and rest intervals [26]. The data were displayed after getting filtered by the inbuilt software.

Urogenital Distress Inventory-6 (UDI-6)

This inventory featured six questions, with responses categorized as "not at all" (0), "a little bit" (1), "moderately" (2), and "greatly" (3). Scores were obtained by summing the responses, dividing by six, and multiplying by 25 to yield the Urogenital Distress Inventory (UDI) score. Scores exceeding 33.33 indicated higher distress [27]. The kappa statistics vary from 0.699 to 0.350 for each question [27]. The sensitivity for UDI-6 is 97% [28].

Statistical analysis

The analysis was carried out after the completion of the 12-week treatment period for all participants. We adhered to the Enhancing the Quality and Transparency of Health Research (EQUATOR) network guidelines for reporting descriptive statistics and tests. This approach accounted for the possibility that dropouts might have returned to their initial readings, and any missing data were replaced with the readings and responses from the first day of assessment. To enhance sensitivity, data available after the 12-week period were also analyzed separately. However, there were no dropouts in the current study.

To explore the differences between the two groups, the Mann-Whitney U-test was utilized. Mean differences at a 95% confidence interval were reported to convey the data. However, for within-group comparison, the Wilcoxon sign rank test was used. All data analyses were conducted by a biostatistician who was blinded to the treatment group assignments. The analysis was performed using IBM SPSS Statistics for Windows, version 23.0 (IBM Corp., Armonk, NY).

Additionally, we calculated the rank-biserial correlation coefficient (r) as a measure of effect size in our study. “r" quantifies the strength and direction of the relationship between groups in a non-parametric context. The interpretation of r is as follows: 0 indicates no relationship, 1 indicates a perfect positive relationship, and -1 indicates a perfect negative relationship. Traditionally, the magnitude of r is considered small if around 0.1, medium if around 0.3, and large if around 0.5 or above. This provides insight into the practical significance of the observed differences between groups. A higher absolute value of r suggests a more substantial effect, and it complements the p-value by quantifying the magnitude of the observed differences [29]. The formula used for the calculation of “r” was r = Z / √N, where, r represents the rank biserial correlation coefficient, and Z is the Z-score obtained from the Mann-Whitney U test. The Z-score is a measure of how many standard deviations an observation or data point is from the mean of a distribution. √N is the square root of the total sample size [29].

## Results

Demographic data analysis conducted with Mann-Whitney U-tests revealed no statistically significant differences between the DNS and Kegel exercise groups in terms of age, height, weight, BMI, number of children, and duration of symptoms (as presented in Table 2).

**Table 2:** Demographics of the participants The median, SD, and range (maximum and minimum values) of the demographics (age, weight, height, BMI, number of children, and duration of symptom) of the DNS and Kegel exercise groups are presented in the table. No statistically significant difference was reported for the demographics as p-value > 0.05. DNS: dynamic neuromuscular stabilization exercise group; Kegel: Kegel exercise group.

	Group	Range		Median	Standard deviation	p-value
		Minimum	Maximum			
Age (years)	DNS	23	40	35	4.9	0.173
	Kegel	25	40	33	3.9	
Weight (kg)	DNS	42	77	60	8.1	0.713
	Kegel	44	75	60	7.81	
Height (cm)	DNS	144	171	154	5.7	0.228
	Kegel	146	168	153	4.67	
BMI (kg/m^2^)	DNS	19.56	30.82	24.9	3.40	0.322
	Kegel	21.08	30.8	26.6	2.8	
Number of children	DNS	0	3	2	0.99	0.735
	Kegel	0	3	2	0.86	
Duration of symptom	DNS	6	48	18	10.25	0.971
	Kegel	6	60	19	12.73	

Strength of the pelvic floor by using a perineometer

Initially, there was no significant difference between the two groups at baseline (p = 0.075). Median ± SD values for DNS and Kegel groups at baseline were 10 ± 2.71 and 10 ± 4.58, respectively. However, at 12 weeks, a statistically significant difference was observed (p = 0.005). Notably, the DNS group exhibited a significant increase in pelvic floor muscle (PFM) strength compared to the Kegel exercise group, i.e., median ± SD values were found to be 24 ± 4.30 and 14 ± 4.60, respectively (Table 3). When comparing each group individually from baseline to 12 weeks, significant improvements were observed in both groups (DNS group: p = 0.005; Kegel exercise group: p = 0.020), as presented in Table 4.

**Table 3:** Results of between-group comparison at baseline and after 12 weeks of intervention Median ± SD values of perineometer in mmHg, EMG (average, peak, and MVC), and UDI-6 at baseline and 12 weeks for DNS and Kegel exercise groups. EMG: electromyography; MVC: maximum voluntary contraction; UDI-6: Urogenital Distress Inventory-6; DNS: dynamic neuromuscular stabilization. * P-value < 0.05 depicts a statistically significant difference between the two groups.

Outcome		Measurements	Group		Median difference (DNS-Kegel)	95% confidence interval (lower limit, upper limit)	p-value for Mann-Whitney U test for between-group comparison	Z-value
			DNS	Kegel				
Perineometer (mmHg)		Baseline (median ± SD)	10 + 2.71	10 + 4.58	-2	-3.35, -0.46	0.075	-1.65
		12 weeks (median ± SD)	24 + 4.3	14 + 4.60	7	6.54, 10.34	0.005*	-3.56
Electromyography	Average (µV)	Baseline (median ± SD)	41.1 + 19.08	37.4 + 16.06	-2	-7, 9.6	0.545	-0.95
		12 weeks (median ± SD)	77.1 + 37.4	55.4 + 18.72	17.6	7.2, 26.8	0.005*	-3.64
	Peak (µV)	Baseline (median ± SD)	78.1 + 43.11	60.2 + 17.2	-10.4	-20.53, 3.22	0.696	-0.40
		12 weeks (median ± SD)	100.3 + 90.7	77.9 + 33.6	21.1	5.48, 32.74	0.001*	-3.45
	Maximum voluntary contraction (%)	Baseline (median ± SD)	50 + 20.8	45.2 + 21.72	-6.8	-11.43, 7.63	0.807	-0.71
		12 weeks (median ± SD)	69.8 + 90.8	58.2 + 16.92	17.8	13.2, 56.18	0.009*	-0.80
UDI-6		Baseline (median ± SD)	42 + 4.95	41 + 4.48	1	-1.37, 2.7	0.481	-0.38
		12 weeks (median ± SD)	14 + 5.45	21 + 7.21	7	3.89, 9.17	0.001*	-0.89

**Table 4:** Results of within-group comparison at baseline and after 12 weeks of intervention Median ± SD of perineometer values, EMG (average, peak, and MVC), and UDI-6 for DNS and Kegel exercise groups at baseline and 12 weeks. EMG: electromyography; MVC: maximum voluntary contraction; UDI-6: Urogenital Distress Inventory-6. * P < 0.05 depicts a significant statistical difference between the baseline and 12 weeks' values for the various outcome measures for the two groups.

Outcome		Group	Measurements		Median difference (baseline-12 weeks)	95% confidence interval (lower limit, upper limit)	P-value for Wilcoxon sign rank test for within-group comparison	Z-value
			Baseline	12 weeks				
Perineometer (mmHg)		DNS	10 + 2.71	24 + 4.3	14	12.07, 14.9	0.005*	-5.84
		Kegel	10 + 4.58	14 + 4.60	3	2.68, 3.58	0.020*	-5.81
Electromyography	Average (µV)	DNS	41.1 + 19.08	77.1 + 37.4	27.7	26.62, 43.29	0.001*	-5.82
		Kegel	37.4 + 16.06	55.4 + 18.72	16.7	13.96, 19.32	0.005*	-5.74
	Peak (µV)	DNS	78.1 + 43.11	100.3 + 90.7	21.2	20.95, 30.65	0.001*	-5.84
		Kegel	60.2 + 17.2	77.9 +33.6	12.3	11.76, 18.90	0.001*	-5.87
	Maximum voluntary contraction (%)	DNS	50 + 20.8	69.8 +90.8	23.1	15.69, 28.51	0.030*	-4.88
		Kegel	45.2 + 21.72	58.2 + 16.92	13	6.34, 14.27	0.005*	-5.32
UDI-6		DNS	42 + 4.95	14 + 5.45	26	23.2, 27.5	0.032*	-5.84
		Kegel	41 + 4.48	21 + 7.21	19	15.68, 20.58	0.046*	-5.87

Electromyography of the pelvic floor muscles

All EMG data were automatically filtered by the inbuilt software of the NeuroTrac MyoPlus2A version 11.1. At baseline, no statistically significant difference was found between the groups for EMG average, EMG peak, and EMG MVC with p-values of 0.545, 0.696, and 0.807, respectively. Median ± SD values for EMG average, EMG peak, and EMG MVC for DNS and Kegel exercise groups at baseline were 41.1 ± 19.08, 78.1 ± 43.11, 100.30 ± 90.7 and 37.4 ± 16.06, 60.2 ± 17.2, 45.2 ± 21.72, respectively. However, a statistically significant difference was observed at 12 weeks for EMG average, EMG peak, and EMG MVC (average p = 0.005; peak p = 0.001; MVC p = 0.009). Significant improvements were noted in both groups for all three components (EMG average, EMG peak, and MVC) when compared from baseline to 12 weeks (DNS group: average p = 0.001; peak p = 0.001; MVC p = 0.030; Kegel exercise group: average p = 0.005; peak p = 0.001; MVC p = 0.005), as presented in Tables 3, 4 for between and within-group analyses, respectively.

UDI-6

No statistically significant difference was found between the two groups at baseline (p = 0.481). Median ± SD values for DNS and Kegel exercise group at baseline were 42 ± 4.95 and 41 ± 4.48, respectively. However, a statistically significant difference was observed between the two groups at 12 weeks (p = 0.001) with median ± SD values for DNS and Kegel exercise groups at 12 weeks as 14 ± 5.45 and 21 ± 7.21, respectively. When evaluating improvements in both groups individually from baseline to 12 weeks, statistically significant improvements were seen in both groups (DNS group: p = 0.032; Kegel exercise group: p = 0.046), as detailed in Tables 3, 4 for between and within-group analyses.

Calculation of “r” effect size

In our study, the "r" values were as follows: 0.72 for perineometer, 0.70 for EMG average, 0.74 for EMG peak, 0.8 for EMG MVC, and 0.89 for UDI-6.

These values indicate that all variables had "r" values greater than 0.7. An effect size of "r" value above 0.7 signifies a medium to large effect size [28]. This suggests that women in the DNS group experienced a more effective treatment compared to the Kegel exercise group for all the variables (Table 5).

**Table 5:** Values of effect size "r" for perineometer, EMG, and UDI-6 EMG: electromyography; MVC: maximum voluntary contraction; UDI-6: Urogenital Distress Inventory-6.

	Outcome measure	Value of "r"
1	Perineometer	0.72
2	EMG average	0.70
3	EMG peak	0.74
4	MVC	0.8
5	UDI-6	0.89

## Discussion

In this study, we conducted a thorough evaluation to assess the relative effectiveness of DNS exercises in comparison to conventional Kegel exercises for the management of SUI in women. Our study is a significant addition to the existing body of knowledge regarding the array of treatment approaches available for addressing SUI, shedding light on potentially more efficient interventions for this widespread condition.

Our investigation revealed substantial improvements across various key parameters following a 12-week intervention with DNS exercises. These improvements included enhanced PFM strength, increased electrical activity in the PFM, and lower scores on the UDI-6. Conversely, the group that underwent traditional Kegel exercises did not exhibit similar significant improvements when compared to the DNS group, particularly in terms of PFM strength, pelvic floor EMG activity, and UDI-6 scores. These findings suggest that DNS exercises may hold promise as a more advantageous intervention, especially for women dealing with SUI.

One of the intriguing aspects of our study is the direct comparison of DNS exercises and Kegel exercises. This comparison fills a gap in the literature, as there has been limited research directly comparing these two approaches in the context of SUI. Our study provides valuable insights into whether DNS exercises, designed to target the integrated spinal stabilization system, including the pelvic floor, present a more effective alternative to the commonly recommended Kegel exercises.

The rationale behind the superior outcomes observed in the DNS exercise group likely stems from the biomechanical link between the diaphragm, abdominals, multifidus, and the pelvic floor [5-8]. DNS exercises are structured to activate and coordinate these muscle groups, which constitute an integrated spinal stabilization system [9-12]. This holistic approach emphasizes the importance of these muscles working together efficiently.

Coordination of the diaphragm, abdominal muscles (including the transverse abdominis), multifidus, and pelvic floor is central to the success of DNS exercises [9-12]. These exercises emphasize the coordinated activation of this integrated system to maintain the continence mechanism under stress. Additionally, it has been reported that if one part of the stabilizing system is affected, it impacts the other parts as well [8]. In contrast to previous approaches that predominantly focused on strengthening individual muscles, DNS recognizes the importance of these muscles acting in unison.

The literature also supports the idea that treating the lumbopelvic system as a whole is crucial for effective SUI rehabilitation [4,6,7]. Another study illustrated that the training of the diaphragm and abdominal muscles along with the pelvic floor was superior to the isolated contraction of PFM for urinary incontinence [24]. The DNS approach, which targets this entire system, has been shown to lead to more significant improvements in our study. It is worth noting that while some studies have focused on different conditions, such as enhancing core stability in stroke patients, they have highlighted the potential of DNS exercises to enhance core stability [9-11]. Core stability, in turn, plays a pivotal role in providing support to the pelvic floor [5-7].

The timeline for observing improvements in our study was 12 weeks, which aligns with the existing consensus in muscle physiology [24]. Previous research has demonstrated that improvements in pelvic floor strength can be observed within a similar timeframe, validating our findings and underscoring the potential for relatively short-term interventions to yield meaningful results [24].

Limitations

It is important to acknowledge the limitations of our study. We did not include a follow-up to assess the long-term effectiveness of the treatment. Our study was single-blinded, and it is essential to consider that the age group of participants was limited to females between 18 and 40 years old, which may affect the generalizability of our results to other age groups. Additionally, participants with severe SUI were not included in the study, restricting the use of DNS exercises to mild to moderate SUI cases. Beyond these considerations, our study did not incorporate an examination of various pertinent factors, including socioeconomic status, the presence of endometriosis, and dietary habits. These omissions represent a potential source of confounding that could influence the observed outcomes. Consequently, the broader applicability of our findings to diverse demographic and health contexts is constrained. Addressing these limitations necessitates future research endeavors incorporating a follow-up mechanism, diverse age groups, and a more inclusive participant selection process. Additionally, a comprehensive exploration of confounding factors and their potential impact on treatment outcomes will contribute to a more nuanced understanding of the intervention's efficacy.

## Conclusions

In conclusion, our study's results suggest that DNS exercises, which emphasize the coordinated activation of the diaphragm, abdominals, multifidus, and pelvic floor, may offer a more effective approach for managing stress urinary incontinence in women compared to traditional Kegel exercises. The biomechanical synergy of these muscle groups appears to be a critical factor in the observed improvements. However, further research is warranted to explore the long-term effectiveness and specificity of this approach for different populations by incontinence type, severity, age, and other important factors. Nonetheless, our study opens new avenues for the management of SUI, offering individuals and healthcare professionals a potentially more effective intervention to consider.

## References

[1] Abrams P, Cardozo L, Fall M (2003). The standardisation of terminology in lower urinary tract function: report from the standardisation sub-committee of the International Continence Society. Urology.

[2] Yip SO, Dick MA, McPencow AM, Martin DK, Ciarleglio MM, Erekson EA (2013). The association between urinary and fecal incontinence and social isolation in older women. Am J Obstet Gynecol.

[3] Kumari S, Jain V, Mandal AK, Singh A (2008). Behavioral therapy for urinary incontinence in India. Int J Gynaecol Obstet.

[4] Grewar H, McLean L (2008). The integrated continence system: a manual therapy approach to the treatment of stress urinary incontinence. Man Ther.

[5] Hodges PW, Sapsford R, Pengel LH (2007). Postural and respiratory functions of the pelvic floor muscles. Neurourol Urodyn.

[6] Sapsford R (2011). The pelvic floor: a clinical model for function and rehabilitation. Physiotherapy.

[7] Sapsford RR, Hodges PW, Richardson CA, Cooper DH, Markwell SJ, Jull GA (2001). Co-activation of the abdominal and pelvic floor muscles during voluntary exercises. Neurourol Urodyn.

[8] Frank C, Kobesova A, Kolar P (2013). Dynamic neuromuscular stabilization & sports rehabilitation. Int J Sports Phys Ther.

[9] Son MS, Jung DH, You JS, Yi CH, Jeon HS, Cha YJ (2017). Effects of dynamic neuromuscular stabilization on diaphragm movement, postural control, balance and gait performance in cerebral palsy. NeuroRehabilitation.

[10] Kim DH, An DH, Yoo WG (2017). Effects of 4 weeks of dynamic neuromuscular stabilization training on balance and gait performance in an adolescent with spastic hemiparetic cerebral palsy. J Phys Ther Sci.

[11] Raghumahanti R, Chitkara E, Agarwal PR (2022). Effectiveness of dynamic neuromuscular stabilisation for improving trunk control in hemiplegic stroke: a scoping mini review. Neurosci Res Notes.

[12] Kobesova A, Kolar P (2014). Developmental kinesiology: three levels of motor control in the assessment and treatment of the motor system. J Bodyw Mov Ther.

[13] Cissik JM (2011). The role of core training in athletic performance, injury prevention, and injury treatment. Strength Cond J.

[14] Warren L, Baker R, Nasypany A, Seegmiller J (2014). Core concepts: understanding the complexity of the spinal stabilizing systems in local and global injury prevention and treatment. Int J Athl Ther Train.

[15] Kobesova A, Valouchova P, Kolar P (2014). Dynamic neuromuscular stabilization: exercises based on developmental kinesiology models. Functional Training Handbook.

[16] Kobesova A, Safarova M, Kolar P Dynamic neuromuscular stabilization: exercise in developmental positions to achieve spinal stability and functional joint centration. Oxford Textbook of Musculoskeletal Medicine.

[17] Davidek P, Andel R, Kobesova A (2018). Influence of dynamic neuromuscular stabilization approach on maximum kayak paddling force. J Hum Kinet.

[18] Agrawal D, Chaudhary V, Raghumahanti R (2021). Effectiveness of dynamic neuromuscular stabilization and motor relearning programme on lumbo pelvic stability in subjects with hemiplegic stroke. Indian J Health Sci Care.

[19] Nisha Nisha, Chaudhary V, Raghumahanti R (2021). Effect of dynamic neuromuscular stabilization (DNS) And modified constraint-induced movement therapy (MCIMT) On trunk and upper limb function in hemiplegic stroke. Indian J Health Sci Care.

[20] Oppelt M, Juehring D, Sorgenfrey G, Harvey PJ, Larkin-Thier SM (2014). A case study utilizing spinal manipulation and dynamic neuromuscular stabilization care to enhance function of a post cerebrovascular accident patient. J Bodyw Mov Ther.

[21] Juehring DD, Barber MR (2011). A case study utilizing Vojta/dynamic neuromuscular stabilization therapy to control symptoms of a chronic migraine sufferer. J Bodyw Mov Ther.

[22] Sharma K, Yadav A (2020). Dynamic neuromuscular stabilization- a narrative review. Int J Health Sci Res.

[23] Julious SA (2005). Sample size of 12 per group rule of thumb for a pilot study. Pharm Stat.

[24] Hung HC, Hsiao SM, Chih SY, Lin HH, Tsauo JY (2010). An alternative intervention for urinary incontinence: retraining diaphragmatic, deep abdominal and pelvic floor muscle coordinated function. Man Ther.

[25] Rahmani N, Mohseni-Bandpei MA (2011). Application of perineometer in the assessment of pelvic floor muscle strength and endurance: a reliability study. J Bodyw Mov Ther.

[26] Bocardi DA, Pereira-Baldon VS, Ferreira CH, Avila MA, Beleza AC, Driusso P (2018). Pelvic floor muscle function and EMG in nulliparous women of different ages: a cross-sectional study. Climacteric.

[27] Skorupska K, Grzybowska ME, Kubik-Komar A, Rechberger T, Miotla P (2021). Identification of the Urogenital Distress Inventory-6 and the Incontinence Impact Questionnaire-7 cutoff scores in urinary incontinent women. Health Qual Life Outcomes.

[28] Hagen S, Hanley J, Capewell A (2002). Test-retest reliability, validity, and sensitivity to change of the urogenital distress inventory and the incontinence impact questionnaire. Neurourol Urodyn.

[29] Berry KJ, Johnston JE, Mielke PW (2018). The Measurement of Association: A Permutation Statistical Approach. Springer.

